# Supplementary material for: A Critical View of the Application of the APEX Software (Aqueous Photochemistry of Environmentally-Occurring Xenobiotics) to Predict Photoreaction Kinetics in Surface Freshwaters
Source: Molecules. 2019 Dec 18;25(1):9. doi: 10.3390/molecules25010009 (PMC7017383; doi:10.3390/molecules25010009)
Supplement: Supplementary file 1 [file molecules-25-00009-s001.zip › 3.2.4_gcc-4.4.0.zip!/3.2.4_gcc-4.4.0/3.2.4_gcc-4.4.0/doc/octave/html/interpreter/Environment-Variables.html]

Environment Variables - Untitled


Next: Current Working Directory,
Previous: Process ID Information,
Up: System Utilities


---

### 34.7 Environment Variables

— Built-in Function: **getenv** (var)  
> Return the value of the environment variable var. For example,
>
> ```
>           getenv ("PATH")
> ```
>
> returns a string containing the value of your path.

— Built-in Function: **putenv** (var, value)  
— Built-in Function: **setenv** (var, value)  
> Set the value of the environment variable var to value.
